# Machine Learning–Based Personalized Prediction of Hepatocellular Carcinoma Recurrence After Radiofrequency Ablation

**DOI:** 10.1016/j.gastha.2021.09.003

**Published:** 2022-02-03

**Authors:** Masaya Sato, Ryosuke Tateishi, Makoto Moriyama, Tsuyoshi Fukumoto, Tomoharu Yamada, Ryo Nakagomi, Mizuki Nishibatake Kinoshita, Takuma Nakatsuka, Tatsuya Minami, Koji Uchino, Kenichiro Enooku, Hayato Nakagawa, Shuichiro Shiina, Kota Ninomiya, Satoshi Kodera, Yutaka Yatomi, Kazuhiko Koike

**Affiliations:** 1 Department of Clinical Laboratory Medicine, Graduate School of Medicine, The University of Tokyo, Tokyo, Japan; 2 Department of Gastroenterology, Graduate School of Medicine, The University of Tokyo, Tokyo, Japan; 3 Department of Gastroenterology, Juntendo University, Tokyo, Japan; 4 Department of Cardiovascular Medicine, Graduate School of Medicine, The University of Tokyo, Tokyo, Japan

**Keywords:** Hepatocellular Carcinoma, Radiofrequency Ablation, Machine Learning, DeepSurv

## Abstract

**Background and Aims:**

Radiofrequency ablation (RFA) is a widely accepted, minimally invasive treatment for hepatocellular carcinoma (HCC). This study aimed to develop a machine learning (ML) model to predict the risk of HCC recurrence after RFA treatment for individual patients.

**Methods:**

We included a total of 1778 patients with treatment-naïve HCC who underwent RFA. The cumulative probability of overall recurrence after the initial RFA treatment was 78.9% and 88.0% at 5 and 10 years, respectively. We developed a conventional Cox proportional hazard model and 6 ML models—including the deep learning–based DeepSurv model. Model performance was evaluated using Harrel’s c-index and was validated externally using the split-sample method.

**Results:**

The gradient boosting decision tree (GBDT) model achieved the best performance with a c-index of 0.67 from external validation, and it showed a high discriminative ability in stratifying the external validation sample into 2, 3, and 4 different risk groups (*P* < .001 among all risk groups). The c-index of DeepSurv was 0.64. In order of significance, the tumor number, serum albumin level, and des-gamma-carboxyprothrombin level were the most important variables for the prediction of HCC recurrence in the GBDT model. Also, the current GBDT model enabled the output of a personalized cumulative recurrence prediction curve for each patient.

**Conclusion:**

We developed a novel ML model for the personalized risk prediction of HCC recurrence after RFA treatment. The current model may lead to the personalization of effective follow-up strategies after RFA treatment according to the risk stratification of HCC recurrence.

## Introduction

Hepatocellular carcinoma (HCC) is the second most common cause of cancer-related deaths worldwide, and its incidence is expected to rise further.^1^ Only 20% of patients with HCC are candidates for resection.^2^ Image-guided minimally invasive techniques have been widely used for the treatment of HCC in the past 2 decades, and radiofrequency ablation (RFA) has yielded promising clinical results, with survival rates comparable with those of hepatectomy.^3^ During the last decade, there has been growing interest in the use of RFA in patients with liver tumors considered to be unresectable owing to impaired hepatic functions or associated comorbidities.^4^^,^^5^ Improvement of surveillance programs—that have reduced the number of HCC cases detected at an already advanced stage—has also increased the use of RFA.^2^

As many as 70% of patients have HCC recurrence within 5 years after RFA treatment; this includes distant recurrence due to intrahepatic metastasis or de novo primary cancer development and local tumor progression.^3^ The HCC recurrence risk varied widely among patients and was shown to be affected by tumor factors—including tumor size, tumor number, or tumor markers—as well as underlying chronic liver diseases—such as fibrosis or inflammation. These contribute to the development of HCC.^3^^,^^6^ Although current guidelines recommend regular follow-ups—using imaging and serum tumor marker studies of patients who received curative treatment for HCC^7^, ^8^, ^9^, ^10^, ^11^, ^12^—surveillance intervals after RFA is preferred to be customized based on the recurrence risk. Objective individual risk assessments and stratifications are warranted for the establishment of personalized surveillance strategies.

Machine learning (ML) is a multidisciplinary field that draws on the fields of computer science and mathematics for developing and implementing computer algorithms, which can maximize predictive accuracy through the development of probabilistic or statistical models, based on existing data.^13^ Deep learning (DL) has been a major topic and has achieved great success in various fields—especially in the field of image recognition.^14^ Also recently; nonlinear ML algorithms such as DeepSurv, a DL generalization of the Cox proportional hazard (CPH) model which can handle survival or cumulative incidence, were developed.^15^, ^16^, ^17^ Although these models have been implemented successfully in the survival or cumulative hazard analysis of various diseases,^18^^,^^19^ there have been no reports found on HCC recurrence prediction after RFA using ML-based survival or cumulative incidence models.

This study aimed to develop an ML model for hazard identification to predict the risk of HCC recurrence after RFA treatment for individual patients. This may lead to personalized settings of RFA follow-up intervals.

## Materials and Methods

### Patients

From February 1999 to December 2019, 1855 patients with treatment-naïve HCC underwent RFA as an initial treatment at the Department of Gastroenterology, the University of Tokyo Hospital. For all 1855 patients, information on patient age at initial treatment; sex; levels of serum albumin, total bilirubin (TB), aspartate aminotransferase (AST), alanine aminotransferase (ALT), serum creatinine, and hepatitis B surface (HBs) antigen; platelet count; prothrombin time (PT); and hepatitis C virus (HCV) antibody status were available. Finally, we enrolled 1778 patients in the present study after excluding 49 patients in whom RFA was performed with a noncurative intent to reduce tumor burden. Owing to warfarin administration, information on serum des-gamma-carboxyprothrombin (DCP) levels regarding 28 patients was not available. The inclusion criteria for RFA were as follows: platelet count of no less than 50 × 10^3^/mm^3^, TB level < 3 mg/dL, and prothrombin activity level > 50%. Patients with macroscopic vascular invasion, refractory ascites, or extrahepatic metastasis were excluded. The clinical data on each patient who underwent RFA in our department were stored in a database designed and maintained prospectively to assess the short- and long-term efficacy and safety of the RFA treatment.

The current study was conducted in accordance with the ethical guidelines for epidemiological research provided by the Japanese Ministry of Education, Culture, Sports, Science and Technology and the Ministry of Health, Labor, and Welfare. The study design was approved by the University of Tokyo Medical Research Center Ethics Committee (Registration No. 2058).

### Diagnosis of HCC

HCC was diagnosed using dynamic computed tomography (CT) or magnetic resonance imaging (MRI); hyperattenuation during the arterial phase and washout during the late phase was regarded as a definite sign of HCC.^20^ When a definite diagnosis of HCC could not be made using CT, an ultrasound-guided tumor biopsy was performed and the pathological diagnosis was based on the Edmondson-Steiner criteria.^21^

### RFA Procedure and Follow-Up

A session was defined as a single intervention episode that consisted of one or more ablations performed on one or more tumors, and the RFA procedure was defined as the completed effort to ablate all tumors that consisted of one or more sessions.^22^ The details of the RFA technique are described elsewhere.^23^ Briefly, RFA was performed under real ultrasound guidance using a single needle pass (Cool-tip®, Covidien, Boulder, CO, USA/Medtronic, Minneapolis, MN, USA, or VIVARF, STARmed®, Gyeonggi-do, Korea). After treatment, we monitored HCC recurrence using dynamic CT or MRI every 4 months. Furthermore, measurements of the serum alpha-fetoprotein (AFP), DCP, and Lens culinaris agglutinin-reactive fraction of AFP (AFP-L3) levels were also monitored. HCC recurrence was diagnosed using the same criteria as applied to the diagnosis of HCC. Liver biochemistry tests were also performed every 4 months to evaluate liver function.

### Model Development

We developed a conventional CPH model and 6 ML models: a DL-based model (DeepSurv), neural multitask logistic regression model (MTLR), random forest (RF), gradient boosting decision tree (GBDT), elastic net penalized regression, and support vector machine (SVM). For model development, we used the following 21 candidate variables: patient age; sex; body mass index; alcoholic consumption; tumor number; maximum tumor size; levels of serum albumin, TB, AST, ALT, serum creatinine, AFP, DCM, AFP-L3, and HBs antigen; platelet count and PT; presence or absence of ascites and encephalopathy; HCV antibody status; and the number of RFA sessions required for treatment. A glossary of terms in the ML models is shown in Table A1.

DeepSurv is a multilayer feedforward network that is inspired by the biological nervous systems for information processing, and it predicts the effects of variables on their hazard rate parameterized by the weight of the network.^17^

The MTLR model is an ML method for survival prediction which is an alternative to the CPH model. This model casts the outcome as a multitask binary classification on a discretized time axis and uses a sequence of dependent logistic regressions to ensure consistency of predictions.^24^ The neural MTLR is an approach for survival prediction based on a neural network and is an extension of the MTLR framework.^25^

Random forests and GBDTs are decision tree (DT) algorithms and are structures for classification. They include a root node, branches, and leaf nodes. RF and GBDT algorithms are ensembles of multiple DT models through bagging and boosting, respectively.^26^ RFs build multiple DTs in parallel, whereas GBDTs build a sequential series of trees—where each tree tries to complement each other and correct for the residuals in the predictions made by all previous trees.^27^^,^^28^ Elastic net penalized regression is a hybrid of the 2 approaches; it has a penalty structure that is a mixture of the LASSO^29^ and ridge^30^ penalties, controlled by a specified mixing parameter.^31^

SVM, a supervised ML technique, is an optimal margin-based algorithm,^32^ which transforms the original data into a higher dimensional space using nonlinear mapping. In the new higher dimension, the SVM finds the appropriate hyperplane which separates the original data according to an annotation profile with the help of support vectors.^33^ All models were implemented in Python using PySurvival^34^ and scikit-survival.^35^ The latter builds directly on the scikit-learn library to implement survival models.

### Statistical Analysis

Continuous variables were expressed as medians with the first and third quartiles, while categorical variables were expressed as numbers and frequencies (%). To evaluate the predictive performance of the model, we randomly split a total of 1778 patients into a training set (90% of patients), which was used to build the model, and a test set (10% of patients), which was used to evaluate the performance of each model. The discrimination performances among the models in both the training set (internal validation) and test set (external validation) were assessed using Harrell`s c-index.^36^ The variable importance for class discrimination in the DT predictive model was assessed using the permutation-based variable importance scores^37^ The discriminative ability of each model was assessed using Kaplan-Meier curves and log-rank tests for various risk groups using R3.6.3 (http://www.R-project.org).^38^ The threshold of *P*-values for significance was set at <.05.

## Results

### Patient Characteristics

We included a total of 1778 patients with treatment-naïve HCC who received RFA as initial treatment at our institution. Table 1 shows the clinical characteristics of the patients. Approximately 60% were male, and the median age was 70 years. The median maximum tumor size and tumor number are 2.2 cm and 1, respectively. The cumulative probability of overall recurrence after the initial RFA treatment was 78.9% and 88.0% at 5 and 10 years, respectively.

**Table 1:** Baseline Characteristics of the 1778 Patients Undergoing Radiofrequency Ablation for Primary Hepatocellular Carcinoma

Variable	Value (n = 1778)
Sex, n (%)	
Female	670 (37.7)
Male	1108 (62.3)
Age (y)	70 (64–76)
Body mass index	23.4 (21.3–36.7)
Viral infection, n (%)	
HBs antigen positive	200 (11.2)
HCV antibody positive	1219 (68.7)
Both positive	15 (0.8)
Both negative	344 (19.3)
Alcoholic consumption, n (%)	
<50 g/d	1346 (75.7)
50–80 g/d	171 (9.6)
>80 g/d	261 (14.7)
Albumin (g/dL)	3.7 (3.4–4.0)
Creatinine (mg/dL)	0.7 (0.6–0.9)
Total bilirubin (mg/dL)	0.8 (0.6–1.1)
Prothrombin time (%)	86 (74–100)
Platelet count (×10^3^/mm^3^)	110 (79–150)
AST (IU/l)	50 (34.25–70.75)
ALT (IU/l)	42 (27–66)
Tumor size (cm)	2.2 (1.7–2.9)
Tumor number	1 (1–2)
Serum AFP (ng/dL)	15 (6–56)
Serum DCP (mAU/mL)	22 (15–49)
Serum AFP-L3 (%)	0.5 (0.5–6.8)
Ascites, n (%)	
Absent	1563 (87.9)
Present	215 (12.1)
Encephalopathy, n (%)	
Absent	1741 (97.9)
Present	37 (2.1)
RFA sessions required for treatment, n (%)	
1	1028 (57.8)
2	590 (33.2)
3 or more	160 (9.0)

Data are expressed as median (1–3^rd^ quartile).

### Comparison of the Predictive Performance of Each Model

The discriminatory performance of the predictive models assessed using Harrell’s c-index is shown in Table 2. The c-index of the CPH model in the test set was 0.64. Among all models, the GBDT model showed the highest c-index of 0.67 (95% confidence interval, 0.61–0.72) in the test set (external validation.)

**Table 2:** Predictive Performance (c-Index) of the Models

Machine learning model	Training set (internal validation)	Test set (external validation)
Cox proportional hazard model	0.64	0.64
DeepSurv	0.67	0.65
Neural multi-task logistic regression model	0.62	0.63
Random forest model	0.74	0.66
Gradient boosting model	0.72	0.67
Elastic net penalized regression	0.62	0.62
Support vector machine	0.63	0.65

### Predictive Models for HCC Recurrence Using the GBDT Model

**Figure 1:**
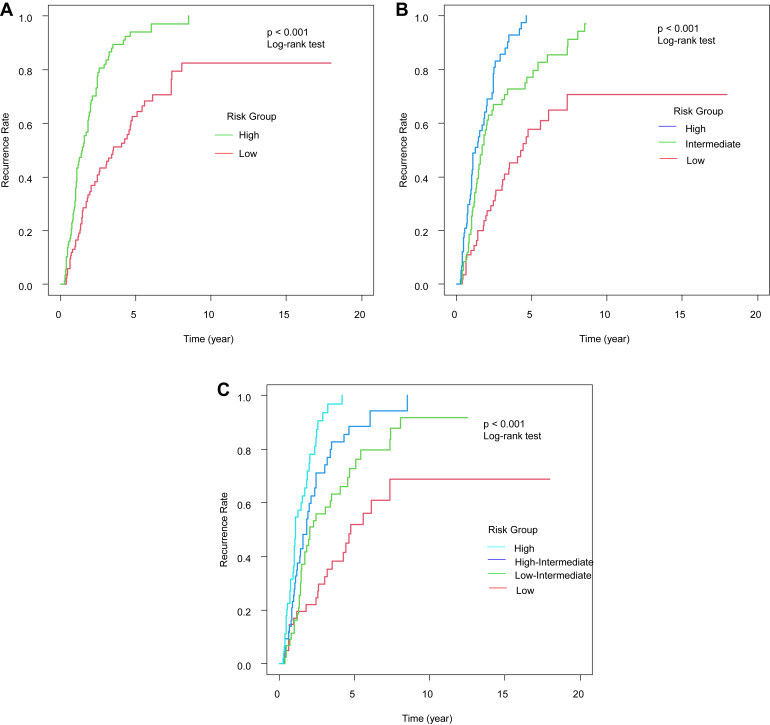
Kaplan-Meier curves for different risk groups of HCC recurrence after initial RFA—categorized into 2 (A), 3 (B), and 4 (C) risk groups using the gradient boosting model.

**Figure 2:**
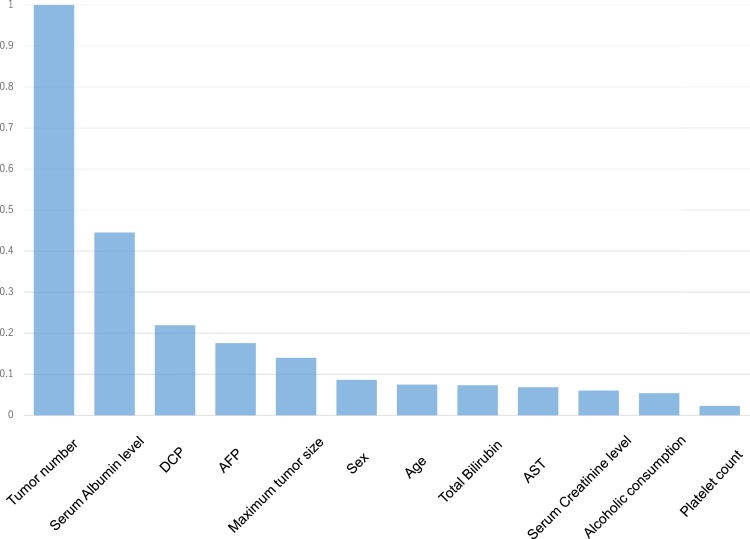
Relative variable importance of the gradient boosting model for the prediction of HCC recurrence assessed using the permutation-based variable importance scores.

Figure 1 shows the Kaplan-Meier curves of HCC recurrence after the initial RFA; they are categorized into 2 (Figure 1A), 3 (Figure 1B), and 4 (Figure 1C) risk groups in the test set using the GBDT model—which is the model that showed the highest c-index among all models. The GBDT model showed high discriminative ability in all different risk divisions (*P* < .001 among risk groups in all risk divisions—see Figure 1A–C). We then investigated the variable importance of the predictive model using the GBDT model developed in the current study. Figure 2 shows the relative importance of each variable assessed using the permutation-based variable importance scores of this model. The most important variables for HCC recurrence were the tumor number, serum albumin level, and DCP level.

### Personalized Prediction of HCC Recurrence in Individual Patients Using the GBDT Model

**Figure 3:**
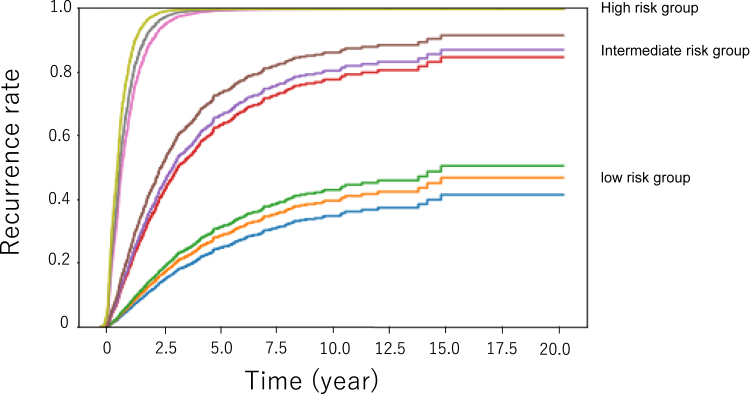
Plots of the predicted cumulative recurrence rate of 178 individual patients in the test set as demonstrated by the gradient boosting model.

**Figure 4:**
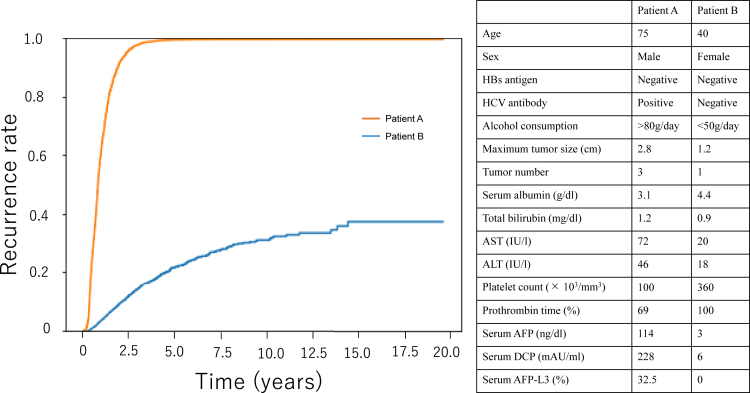
Personalized prediction of HCC recurrence after RFA in 2 hypothetical patients: a high-risk case of recurrence in a man in his 70s with large multiple lesions and high tumor markers (Patient A) and a low-risk case in a woman in her 40s with a small single lesion and normal tumor markers (Patient B). Details of the 2 hypothetical patients are shown in the table on the right in the figure.

Finally, we applied the GBDT model for the prediction of HCC recurrence after RFA in individual patients. Figure 3 shows plots of the cumulative recurrence rate for 9 individual patients, 3 each from the high-, intermediate-, and low-risk groups, in the test set predicted by the GBDT model. The predicted HCC recurrence rate varies widely depending on a variety of factors, including patient background or tumor factors. We also applied the current model to predict HCC recurrence after RFA in 2 hypothetical patients: a high-risk case of recurrence in a man in his 70s with large multiple lesions and high tumor markers (Patient A) and a low-risk case in a woman in her 40s with a small single lesion and normal tumor markers (Patient B) (Figure 4). The GBDT model made a clear distinction between the high-risk Patient A and low-risk Patient B.

## Discussion

In the current study, we developed CPH and ML models to predict the risk of HCC recurrence after an initial RFA treatment. We compared the conventional CPH model based on the linear fitting of variables^39^ and nonlinear ML models. Model fitting is important for a successful predictive method. If the data are linearly separable, a linear model will fit the data.^40^^,^^41^ Variables often have nonlinear effects with cancer recurrence in clinical settings.^42^^,^^43^ In actual fact, the nonlinear GBDT model showed the best predictive performance assessed using the c-index in the present study. The GBDT model showed a higher predictive value compared with DeepSurv which has a DL architecture. Although DeepSurv is a promising and powerful model for the survival or time-to-event prediction,^17^ this model did not always show the best performance for event prediction in the medical field.^19^^,^^44^ Identifying an optimal classifier depending on the available data is important to obtain precise predictions of HCC recurrence.

Each ML model has its strengths or weaknesses. DL has enabled major breakthroughs in the processing of images, video, speech, or audio.^14^ Although DL is a powerful technique, it requires a large polynomial sample size in terms of the depth of the network to obtain ideal convergence boundaries.^45^ This may be difficult to achieve in clinical settings for ethical or methodological reasons. The main advantage of DT models (GBDT and RF) which showed the highest predictive performance in the current study is their capability to break down a complex structure into a collection of simpler structures.^46^ The major advantage of the RF model is that it is robust to noise and overfitting,^47^ whereas those of GBDT models are its state-of-the-art predictive performance on tabular data and the customizability of loss of function.^26^^,^^48^ The elastic net model ensures sparsity in the feature weights and promotes a grouping effect of the highly correlated features.^49^ In addition, the SVM can, with relative ease, overcome the high dimensionality problem (ie, the problem that arises when there is a large number of input variables relative to the number of available observations).^50^ However, the SVM is computationally expensive because it involves quadratic optimization problems.^51^

In the current study, we also analyzed feature importance for the prediction of HCC recurrence using the GBDT model. The tumor number, serum albumin level, and DCP level, which are well-known risk factors for HCC recurrence,^3^^,^^52^ were found to be the most important variables. In line with our results, serum albumin level was shown to be a significant predictor of recurrence or recurrence-free survival after a curative treatment for HCC.^53^, ^54^, ^55^ Huang et al^19^ developed a predictive ML model for HCC recurrence after surgical resection. In this study, the GBDT model was selected as the optimal model having a c-index of 0.697 in the external validation, and tumor factors such as tumor number, tumor size, or vascular invasion were identified as important predictors. Yamashita et al^56^ developed a DL-based system (HCC-SurvNet) that predicts the risk of HCC recurrence using histological digital images of paraffin-embedded HCC samples and demonstrated a c-index of 0.683. In this study, tumor factors such as tumor size and microvascular invasion were shown to be candidate predictors of HCC recurrence, which were significantly associated with the HCC-SurvNet score. Because the treatment options and data sets were different, a quantitative comparison of c-indices may not be practical. The c-index of our model, 0.67, is comparable or slightly lower than the aforementioned models.^19^^,^^56^ Because tumor biopsies are not usually performed in RFA procedures—owing to concerns about the risk of tumor dissemination^57^—we could not obtain the histological information. The lack of inclusion of important factors, such as microvascular invasion, may be one of the probable reasons for the slightly lower predictive performance of our ML model than the models in previous reports.

Personalization is one of the ultimate goals of modern medicine.^58^ To realize personalized medicine, the individualized prediction of risk for each patient is essential. In the current study, we utilized the developed ML model for the individual prediction of HCC recurrence for each patient. To the best of our knowledge, the present study is the first demonstration of the personalized prediction of HCC recurrence—after RFA—in individual patients using an ML model. This may lead to the individualization of follow-up intervals after RFA. However, the accuracy for the prediction of HCC recurrence is not satisfactory in the current study. The accurate predictions of recurrent HCC after curative treatment were limited to fairly poor performances with c-indices less than 0.7^59^ in the current and previous studies using clinical data or histological images of resected tumors.^19^^,^^56^ This is likely because of its biological heterogeneity, variety of recurrent patterns (ie, de novo recurrence, local tumor progression, or intrahepatic metastasis),^3^ or technical complexity of treatment procedures. Because the predictive accuracy of ML models depends on the number of samples for training,^60^ further studies for the improvement of predictive models using larger sample size are needed for the clinical use of such models. The integration of multiple heterogenous information (ie, image modality and clinical data information) using a multimodal representation model ^61^ may be a useful approach to obtain greater predictive performance.

Current guidelines recommend regular follow-ups using imaging and serum tumor markers studies of patients who received curative treatments for HCC.^7^, ^8^, ^9^, ^10^, ^11^, ^12^ The American Association for the Study of Liver Diseases provides a guideline that recommends a 3- to 4-month imaging interval, after initial treatment, of up to 2 years; and thereafter, the interval can be at less frequent intervals.^7^ The European Association for the Study of the Liver and the Japanese Society of Hepatology also recommend 3- to 4-month follow-up intervals according to the surveillance approaches used in extremely high-risk cases at the time of onset.^8^^,^^11^ CT and MRI were used for post-RFA surveillance; therefore, individualization of the follow-up interval according to the risk of recurrence is desirable with regards to radiation exposure and medical economics. Predictive models provide a personalized assessment of the probability of a clinical event using patient- or tumor-specific characteristics—and will be further incorporated into the field of cancer medicine in the future.

The limitation of the current study is its single-center design that limited the generalizability of the findings, and therefore, our predictive model might not be generalizable to other practice settings. Further validation in a multicenter setting is required to confirm the general feasibility of the proposed ML model in the present study.

In conclusion, we developed a novel ML model for the personalized risk prediction of HCC recurrence after RFA treatment. The current model may lead to the personalization of follow-up strategies after RFA treatment according to the risk stratification of HCC recurrence.

## Supplementary Materials

Table A1
