# Supplementary material for: Machine Learning–Based Personalized Prediction of Hepatocellular Carcinoma Recurrence After Radiofrequency Ablation
Source: Gastro Hep Adv. 2022 Feb 3;1(1):29–37. doi: 10.1016/j.gastha.2021.09.003 (PMC11308827; doi:10.1016/j.gastha.2021.09.003)
Supplement: Table A1 [file mmc1.docx]

Supplementary Table 1 Glossary of terms used in the machine learning models

| Terms |  | Definition |
| --- | --- | --- |
| DeepSurv |  | Multi-layer feedforward network (deep learning method) for survival analysis |
| Neural multi-task logistic regression model |  | Deep neural networks for survival analysis based on a multi-task logistic regression, which fits a generalization of the logistic regression model to predict a survival outcome |
| Random forest |  | These are ensembles of multiple decision tree algorithms. Decision trees are built in a parallel manner. |
| Gradient boosting |  | These are ensembles of multiple decision tree algorithms.  Decision trees are built sequentially and learn from the errors of previous trees. |
| Elastic net penalized regression |  | Penalized linear regression analysis technique, which is able to address multicollinearity between the predictors and to select the most important ones. |
| Support vector machine |  | Algorithm for kernel classifiers that identify the optimal middle hyperplane, which induces the largest margin. |
